# The need for new vector control approaches targeting outdoor biting Anopheline malaria vector communities

**DOI:** 10.1186/s13071-020-04170-7

**Published:** 2020-06-10

**Authors:** Seynabou Sougoufara, Emmanuel Chinweuba Ottih, Frederic Tripet

**Affiliations:** Centre of Applied Entomology and Parasitology, School of Life Sciences, Keele University, Staffordshire, UK

**Keywords:** *Anopheles*, Mosquitoes, Pesticide resistance, Exophagy, Exophily, Outdoor biting, Traps

## Abstract

Since the implementation of Roll Back Malaria, the widespread use of insecticide-treated nets (ITNs) and indoor residual spraying (IRS) is thought to have played a major part in the decrease in mortality and morbidity achieved in malaria-endemic regions. In the past decade, resistance to major classes of insecticides recommended for public health has spread across many malaria vector populations. Increasingly, malaria vectors are also showing changes in vector behaviour in response to current indoor chemical vector control interventions. Changes in the time of biting and proportion of indoor biting of major vectors, as well as changes in the species composition of mosquito communities threaten the progress made to control malaria transmission. Outdoor biting mosquito populations contribute to malaria transmission in many parts of sub-Saharan Africa and pose new challenges as they cannot be reliably monitored or controlled using conventional tools. Here, we review existing and novel approaches that may be used to target outdoor communities of malaria vectors. We conclude that scalable tools designed specifically for the control and monitoring of outdoor biting and resting malaria vectors with increasingly complex and dynamic responses to intensifying malaria control interventions are urgently needed. These are crucial for integrated vector management programmes designed to challenge current and future vector populations.
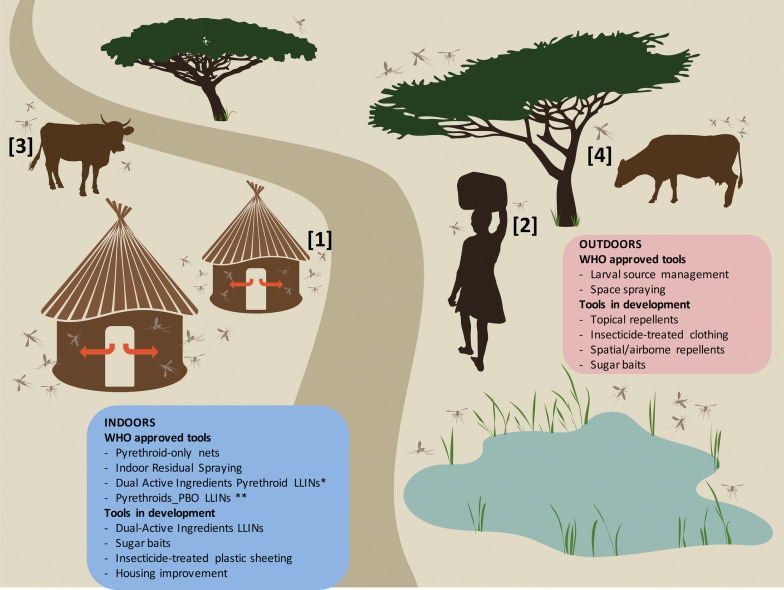

## Background

Despite the substantial gains achieved by the Roll-back Malaria initiative (RBM) since the late 1990s, much of the African continent remains highly endemic for the disease and 93% of malaria deaths occur in this region [1]. Malaria control strategies in sub-Saharan Africa (SSA) rely heavily on programmes targeting vector populations through chemical interventions such as insecticide-treated bednets (ITNs) and indoor residual spraying (IRS). These tools are estimated to have contributed to a 68% and 10% decrease, respectively, of malaria cases since the beginning of their broad-scale implementations in the early 2000s [2]. This progress has brought a number of countries to so-called pre-elimination status, and led the World Health Organization (WHO) and Roll Back Malaria (RBM) to revise their target to the new ambitious goal of reducing the global burden of malaria by 90% by 2030 [3, 4].

**Fig. 1:**
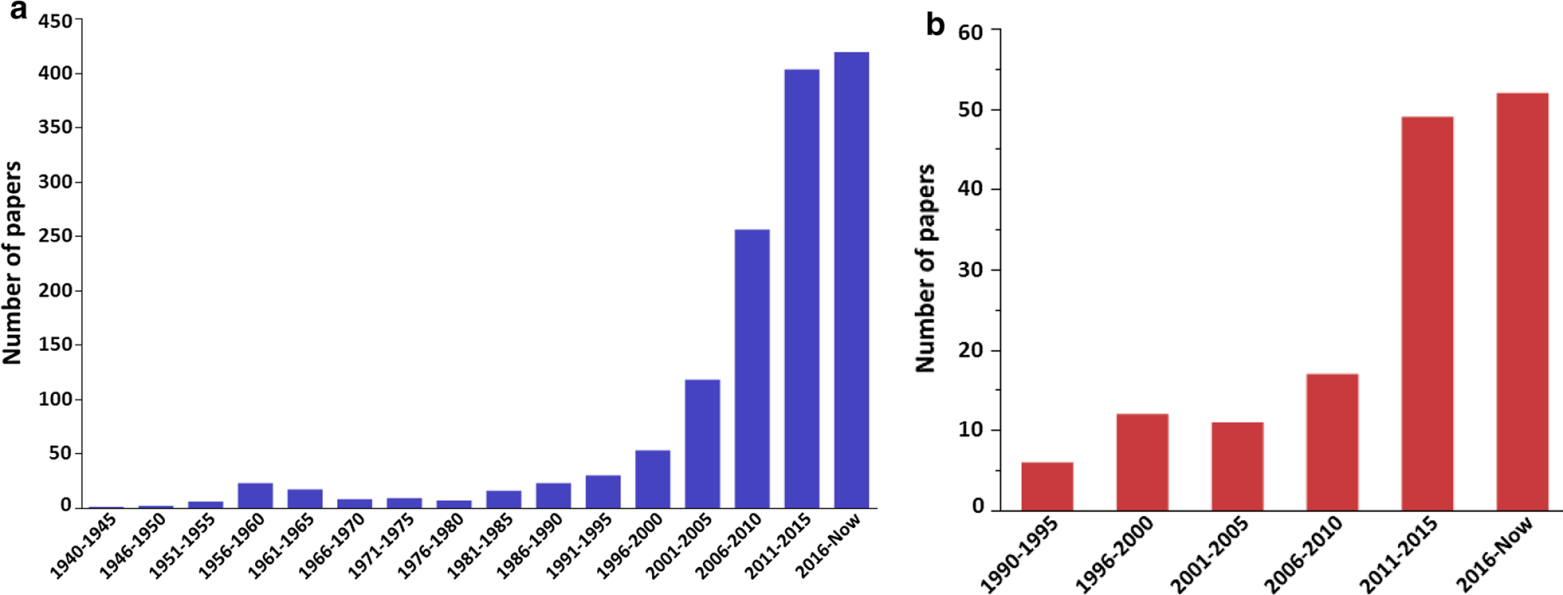
The increasing trend in numbers of peer-reviewed publications focusing on: **a***bednets* or *spraying* and *insecticide resistance* in *anophelines* in *Africa*; and **b** on *outdoor* or *early* or *exophily* and *biting behaviour* in *anophelines* in *Africa* in the online Web of Science database (clarivate.com/webofsciencegroup/solutions/web-of-science/) (Search terms are in italics)

**Fig. 2:**
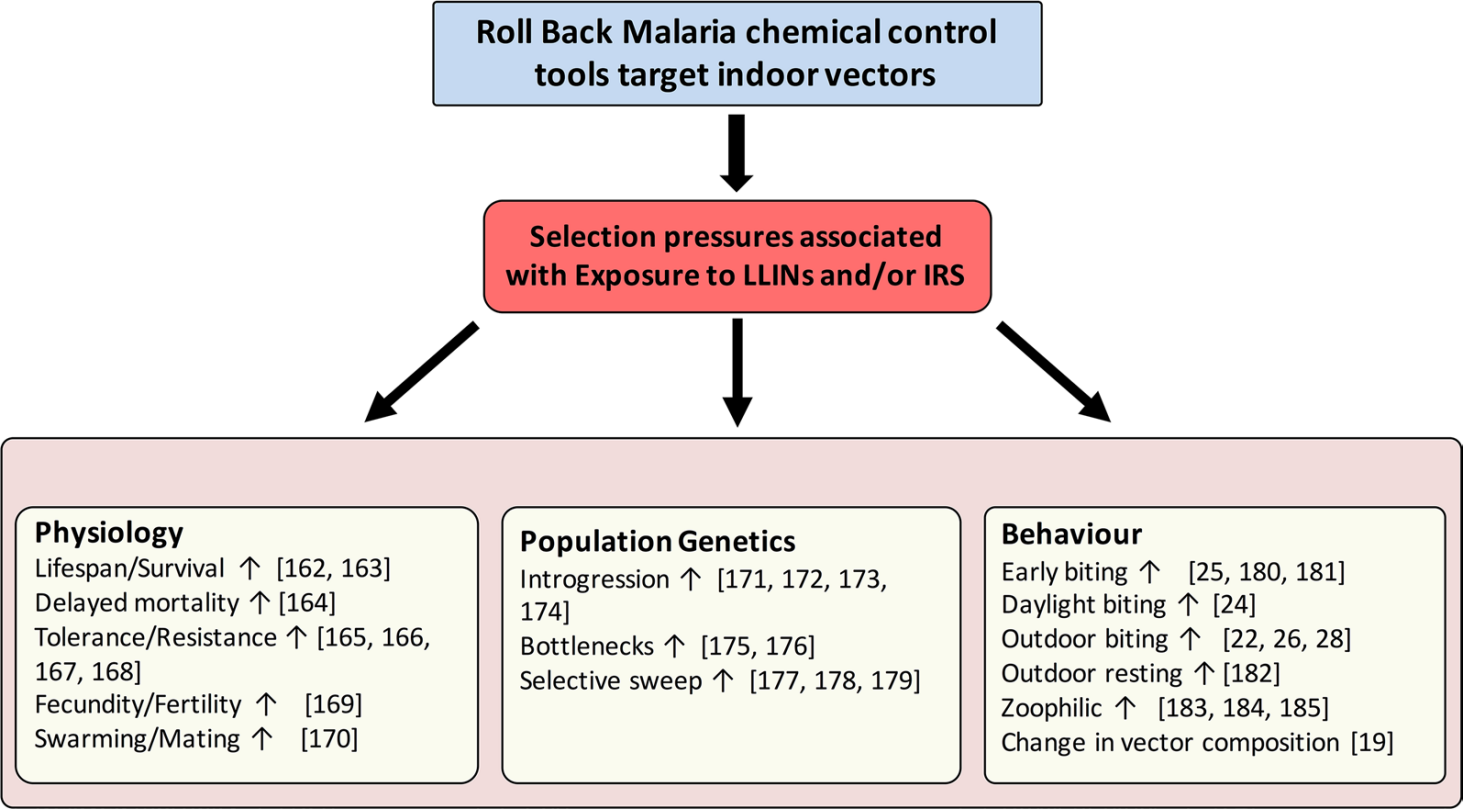
The selective pressures associated with indoor chemical vector control interventions affect many biological characteristics of mosquito populations and mosquito traits that affect vectorial capacity and malaria transmission. Upward arrows denote an increase in the trait considered

Entomological surveillance and monitoring are crucial to the different approaches developed through the WHO Global Technical Strategy towards malaria elimination [3]. Entomological and epidemiological data have highlighted resurgence in malaria transmission in several areas in SSA that had achieved high vector control coverage using ITNs and IRS [5–8]. For a long time, indoor chemical control tools have typically been the most effective against mostly endophagic and endophilic malaria vector species and populations [9]. Unfortunately, the efficacy of these tools is threatened because of the rapid evolution and spread of insecticide resistance in the main malaria vectors in many regions of SSA (Fig. 1a) [10, 11]. Worryingly, other studies have reported that resistant *Anopheles* phenotypes may be more susceptible to *Plasmodium falciparum* infection [12–14] highlighting another risk that could be linked with the escalation of pesticide-based indoor interventions. Beyond the insecticide resistance phenomenon, the selective pressures associated with pesticide exposure affect a large number of mosquito traits including behaviour, genetics, and physiology (Fig. 2). These parameters can affect the vectorial capacity and/or importance of anopheline vectors and are important determinants of local patterns of malaria transmission.

The most efficient malaria vectors in SSA, *Anopheles gambiae*, *Anopheles coluzzii* and some members of the *Anopheles funestus* group exploit larval breeding sites near human habitats and feed preferentially on humans. They are considered to be predominantly endophagic and endophilic [15] but these traits are somewhat plastic and levels of outdoor biting and resting vary between populations. There are also reports of *An. gambiae* (*s.s.*) populations with high levels of exophily that pre-date the intensification of chemical vector control [16, 17]. The sibling species, *Anopheles arabiensis* is known to frequently bite and rest outdoors [18]. In recent years, reports of behavioural shifts observed in response to intensified ITNs and IRS interventions have accumulated suggesting that they play an increasingly important role in malaria resurgence (Fig. 1b). Several studies conducted in SSA showed that *An. arabiensis* has replaced *An. gambiae* (*s.s.*) and *An. coluzzii* as the most dominant species following the intensifications of ITNs use [19–22]. Another study conducted in Kenya showed a shift in vector species with *An. arabiensis* and *An. merus* taking the place of *An. gambiae* (*s.s.*) and *An. funestus* as main malaria vectors [23]. In some regions, these populations now display behavioural avoidance, either through behavioural resilience or the evolution of behavioural resistance, towards indoor control tools such as actively seeking human hosts earlier at dusk and sometimes until dawn, feeding on non-human hosts, and increasingly resting outside. In Senegal, diurnal activity of *An. funestus* has been reported after the introduction of ITNs [24]. Another study in Ethiopia reported early evening activity by *An. arabiensis* with a peak activity between 19 and 20 h after the introduction of ITNs [25]. Earlier biting patterns might be concomitant with outdoor biting activities, as recently reported in Senegal in *An. gambiae* (*s.l.*) and *An. funestus* following two campaigns of ITNs renewal [26]. In Tanzania, *An. arabiensis* and *An. funestus* exhibited outdoor biting patterns, and were active early in the evenings after 47% of ITNs use [22]. Similar patterns were reported from a study testing the efficacy of outdoor landing boxes for anopheline control [27]. The tendency of outdoor biting was also described in the early morning hours in *An. coluzzii* and *An. melas* populations on Bioko Island [28]. This highlights the heterogeneity of *Anopheles* species and the predisposition of some vectors, such as *An. arabiensis*, to feed to an even higher degree outdoors and often on non-human hosts in response to the use of indoor vector control tools [29]. The result is that changes in vector behaviour, whether through resistance or resilience, are currently one of the most important challenges to malaria control, and alternative strategies to tackle outdoor populations at adult and immature stages need to be developed urgently.

Despite growing evidence of the importance of outdoor transmission, most tools for entomological surveillance and monitoring typically focus on indoor mosquito populations and may no longer be adequate for characterising the fast-changing composition and feeding behaviour. The human landing catches (HLC), which has long been the most efficient method of collection for anthropophilic endo- and exophagic vector species, is no longer possible in many regions [30, 31]. This method is based on capturers catching mosquitoes as they land on their exposed legs throughout the night, providing information on the timing of bites by local vector species. Understandably, the use of HLC has now been discouraged on ethical grounds as human-baits may not only be exposed to malaria vectors but, increasingly, to aedine mosquitoes carrying arboviruses for which prophylaxis or treatment is not yet available. Traps commonly used for monitoring indoors such as the Centre for Disease Control and Prevention light traps (CDC-LT) do not perform equally well for outdoor mosquito collections [32–34]. Indoor resting sampling by pyrethroid spray catch (PSC) is a commonly used tool that has no outdoor equivalent. Resting boxes, have long been used for indoors and outdoors monitoring [35] but their effectiveness outdoors varies greatly with the availability of natural resting sites, and seasonal factors, time of day, rainfall and humidity [36].

**Fig. 3:**
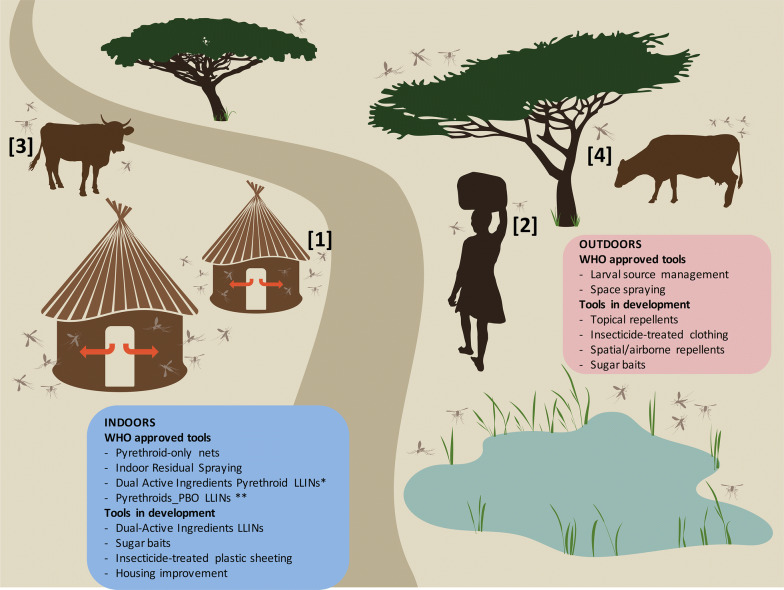
Schematic representation of mosquito distribution in a typical rural habitat. The selective pressure on indoor mosquito populations resulting from the implantation of ITNs and IRS induce behavioural changes of mosquitoes that bite increasingly outdoors (1), earlier at dusk and/or later at dawn when humans are not protected (2). Mosquitoes may also feed more often on non-human hosts (3), and rest outdoors (4) to avoid exposure to vector control. Most WHO-approved tools currently focus on the control of indoor populations (blue boxes) and those that are in development or under interim approval follow the same trend (*), leaving few current options for scalable control of outdoor biting populations (www.who.int/vector-control/vcag/new-interventions/en/). *Interim approval; ** Pyrethroid-PBO net in areas with metabolic resistance

Thus, as is the case for vector control programmes, entomological monitoring surveys require novel sampling approaches and methodologies that address increasingly variable vector population feeding and resting patterns in order to perform effective surveillance and planning of vector control interventions. The paucity of vector control tools approved or under interim approval by WHO or in development targeting outdoor mosquito populations underscores these needs (Fig. 3). The objective of this review is to discuss and highlight tools that may best address the urgent need for outdoor vector population monitoring and control. Existing surveillance and control tools have already been reviewed in the general context of malaria control and elimination elsewhere [37–39]. Consequently, rather than attempting to be exhaustive, we will focus on those relevant to outdoor sampling and discuss in more depth those that are novel and/or scalable tools and could therefore help tackle the emerging challenges posed by the fast evolution of exophagic and exophilic malaria vector communities.

## Traps for host-seeking females

Capturing females as they seek a host to blood-feed and produce eggs is one of most effective ways of sampling mosquito populations. Focussing on this important female life-stage is often preferred as it directly relates to mosquito population demographics as well human exposure to potentially infective bites, hence disease transmission.

### Host-baited traps

Different host-baited traps have been developed to monitor mosquito biting behaviour as a safer alternative to human landing catches (HLC) both indoors or outdoors. Human-baited traps such as the Mbita trap use a volunteer protected by a bednet to attract mosquitoes within a larger netted trap chamber [40], but was not as effective as HLC in collecting outdoor mosquitoes, as revealed by a comparative study conducted in Madagascar [41]. Furvela tent-traps were also developed to collect outdoor mosquitoes. A Furvela trap has a CDC-LT trap (without a light) fixed to the outside of a tent. The tent is occupied by a volunteer whose odour attracts mosquitoes [42]. Other variations are the Ifakara Tent A and B traps which operate by drawing mosquitoes into funnel entrances tilted upward into an upper rectangular section of a canvas tent, the human bait rests in the tent’s lower section protected by netting [31]. Host Decoy Traps (HDT) draw odours from a host housed in a tent and release them through a pipe onto a warm, black sticky target. Depending on the host used, these traps sometimes captured larger number of host-seeking females than HLC [43].

Recently, the mosquito electrocuting trap (MET) was developed as direct replacement to HLC for collecting mosquitoes indoors and outdoors at given time intervals throughout the night. It consists in an electrified square box, in which a human volunteer places his/her legs to attract mosquitoes that get electrocuted upon contact with the box [44]. Promisingly, METs have produced estimates of mosquito biting rates and timing of biting that closely correlate with those produced by HLC [45].

Whilst being valuable monitoring tools, host-baited traps are often large and cumbersome to set-up. Furthermore, their need for hosts makes them intrinsically labour intensive, thereby precluding their use for large-scale vector control programmes.

### Odour-baited traps

Advances in the sensory and chemical ecology of mosquitoes have stimulated the development of a host of novel traps exploiting mosquito attraction to CO_2_, human odours, chemical attractants, and manipulating visual cues [39, 46–48]. In principle, cost-effective traps could provide another angle of attack for controlling both indoor and outdoor vector populations.

In the late 1990s, the development of counter flow geometry (CFG) greatly increased the efficacy of mosquito traps [49]. CFG traps operate by producing a downward flow of air exiting a chemical lure from the trap entrance to attract mosquitoes, an updraft flow then sucks the mosquitoes into a collector [49]. A study conducted in Kenya showed that the addition of attractants such human foot odour and CO_2_ greatly increase the ability of CFG traps to capture *An. gambiae* (*s.s*.) [50]. However, CFG traps with octenol and dry ice were not as effective for collecting *Anopheles* mosquitoes compared to HLC [50, 51]. This highlights the need to further optimize the lures used to attract mosquitoes to CFG traps.

Counter flow technology was exploited in the Biogent Sentinel Trap (BGS) which effectively combines olfactory and visual cues for sampling aedine species and has become a major tool in arbovirus surveillance programmes [52]. The BGS trap uses black and white contrast and a chemical lure which mimics human skin odour [53]. This trap has been evaluated for surveillance of African anopheline malaria vectors. Interestingly, in Burkina Faso, BGS traps baited with BG lure and CO_2_ collected more *An. coluzzii* than CDC traps outdoors during dry and rainy seasons [54]. This same pattern was also observed in Brazil, but placing the trap above ground with a downwards airflow orientation led to higher catch rates of *An. darlingi* than CFG, CDC and the Fay-Prince traps, which were comparable to HLC catches [55]. These results showed that in some settings the so-called BG-Malaria (BGM) inverted BGS trap could potentially be as effective as HLC for monitoring mosquitoes. In semi-field studies conducted in Tanzania, BGM traps were more effective in sampling *An. arabiensis* compared to BGS with or without CO_2_ and synthetic human odours [56]. Additionally, the BG-lure combined with CO_2_ was shown to be more effective than other odour blends [57]. These findings showed that the BGS trap, particularly in its BGM configuration, could be a valuable trap for capturing outdoor African malaria vectors, even when outdoors. However, given the current price-tag of their synthetic lure and their moderate anopheline mosquito catch rate, CFG-based traps would generally benefit from further improvements resulting in increased cost-effectiveness (Table 1).

**Table 1:** Characteristics of representative monitoring and control tools and their potential for scaled-up programmes targeting outdoor biting and resting anopheline mosquito populations in Africa (see text for details)

Tool	Outdoor/indoor surveillance	Compared to HLC	Estimated cost/unit^b^	Scalable for outdoor control	Status of development
BGS and BGM traps	Outdoor [54, 55]	Yes [55, 186]	$100–200	Yes	Commercialised
Mosquito Magnet	Outdoor [59]	Yes [60]	$300–1000	No	Commercialised
Clay pots^a^	Outdoor [67]	No	$1–$50	Yes	Under development
Resting boxes^a^	Indoor and outdoor [35, 66]	Yes [64, 187]	$1–$50	Yes	Under development
Attractive Toxic Sugar Baits	Indoor and outdoor [71, 188]	No	$1–50	Yes	Three RCT protocols reviewed by VCAG
Larvicides	na	na	$1–50	Yes	Commercialised
Genetically modified mosquitoes	na	na	Not available	Potentially	Cage studies results communicated to VCAG
Genetically modified symbionts	na	na	Not available	Potentially	Under development
Endectocides	na	na	$1–50	Yes	RCT protocol in review

^a^And variations and improvements thereof (see text for details)

^b^US dollars

*Abbreviations*: na, not applicable; RCT, randomized control trial; VCAG, Vector Control Advisory Group

Another trap making use of CFG is the mosquito magnet (MM) trap which converts propane gas into CO_2_ and emits heat and moisture to attract mosquitoes [58, 59]. In French Guiana, the MM trap combined with 1-octen-3-ol and HLC, were 2-fold more efficient collectors of anophelines than the CDC Light trap (LT) with or without human bait [60]. Further studies in Tanzania, showed that baiting the MM trap with a worn sock (foot odour) greatly increased its efficacy [61] and that using CO_2_ was crucial, combined with natural or synthetic odours [58–60, 62]. Despite its efficacy as a monitoring tool, the bulk and high cost of the MM trap makes it much less scalable than other alternatives (Table 1).

## Resting traps

A much more affordable option is to target female mosquitoes in search of a resting site after a blood meal. Resting traps offer and opportunity to capture females that are seeking shelter outdoors in a shaded and hidden location whilst digesting the blood meal and maturing their eggs, but they can also capture adult females at other life stages as well as males. Historically, two of the most widely used methods for sampling indoor resting mosquitoes were pyrethrum spray catches (PSC) and aspiration from pit shelters [36], but neither of them are practical or scalable for large-scale outdoor surveillance and vector control.

Resting boxes (RB) are one of the simplest methods used for surveillance and control of mosquitoes outdoors [35]. They are commonly made from cardboard, wood or a dark plastic container and placed near human habitations. RB traps provide artificial shelter against predators, heat and desiccation, thereby attracting blood-fed, semi-gravid and gravid females and also males [35, 63]. In Tanzania, RBs baited with cow urine caught more *An. arabiensis* outdoors than HLC [64]. As expected, they collected more fed, semi-gravid and gravid females of *An. arabiensis* than the CDC light trap method, which caught only unfed host-seeking females [35]. Resting boxes also have the advantage of attracting various species of mosquitoes including *Anopheles*, *Culex* and *Culiseta* mosquitoes [53, 65]. A sticky version of resting boxes (SRB) has also been developed for more efficient trapping in Burkina Faso, where a higher diversity of mosquitoes was collected indoors and outdoors using SRBs when compared to backpack aspiration inside houses (BP) and pit-shelters used outdoors (PIT) [66]. Resting boxes are cheap to make with local materials, easily scalable, and therefore provide another scalable tool for surveillance and control of outdoor and indoor resting mosquitoes alike [35] (Table 1).

African water storage clay pots are another format of resting traps for indoor and outdoor sampling of various mosquito species [67, 68]. In western Kenya, clay pots used outdoors collected a larger number of male and female *An. arabiensis* and *An. gambiae* compared to pit-shelter traps [67]. While in Tanzania, Bijllaardt et al. [68] showed that clay pots used indoors collected a higher proportion of blood-fed females than CDC light traps. Clay pots have also been used in combination with entomophagic fungi for biological control [69]. Exposure to conidia applied to the inside of a resting pot has resulted in the decrease of longevity in both females and males of *An*. *gambiae* and *An. funestus* [69]. Some studies have shown that human odour or animal urine can further improve the attractiveness of clay pots, making them a locally-producible and scalable monitoring tool (Table 1). Some of their drawbacks are their heavy weight and fragility compared to other resting boxes.

There are many variations around the resting box format that can be used both indoors and outdoors. In the outdoor setting, the attractiveness of resting traps to mosquitoes depends on many environmental factors [36]; such as the availability of other resting sites (vegetation, holes and crevices), and harsh weather conditions that encourage mosquitoes to seek shelter (e.g. dry season). This limits their outdoor efficacy to some settings and environmental conditions.

## Attractive toxic sugar baits (ATSB)

The use of ATSB is a promising novel approach targeting sugar feeding, another lesser-known part of the mosquito life-style. Newly emerged mosquitoes need energy reserves for flying, mating and blood feeding [70]. Both males and females draw their nutrient sources by feeding on plant nectar, flowers and fruits to cover their energy needs. For this purpose, the use of ATSB has been explored to attract mosquitoes with fruity and flowery scents combined with sugar solutions and a toxic compound to kill them.

The potential of this novel approach against African malaria vectors was demonstrated in Mali where a single outdoor application of ATSB laced with boric acid resulted in a 90% decrease in *An. gambiae* (*s.l*.) densities [71]. Research efforts have focused on optimizing the dosage of toxic compounds such as eugenol, boric acid, spinosad and dinotefuran to best balance toxic and repellent effects [72, 73]. As an example, an intermediate concentration of 1% of eugenol achieved the highest mortality rates of *An. quadrimaculatus* compared to concentrations 0.1 and 10% [74]. Whilst ATSB has the potential of becoming an affordable and scalable new vector control tool, the attractiveness of the toxic bait to mammals and children is a concern [75]. Thus ivermectin, which is non-toxic to mammals and an effective endectocide, was successfully used to control semi-field cage populations of *An. arabiensis* resulting in a 95% decrease in 48 h [76]. Whilst mathematical models suggest that ATSBs can have strong effects on malaria transmission, particularly because of their effect on female lifespan [77], several issues are currently limiting their deployment. In the context of widespread resistance to common chemical control interventions in anopheline vectors, the possible evolution of resistance to toxic bait compounds and interactions with existing resistant mosquito phenotypes needs to be considered. Of particular relevance is the use of oral toxins such as boric acid, tolfenpyrad and chlorfenapyr whose mode of actions contrasts with that of neurotoxic insecticides and were shown to be effective against populations of *An. arabiensis* and *Culex quinquefasciatus* resistant to pyrethroids [78]. Another current concern associated with ATSB deployment is their potential detrimental effect on non-target insects, particularly when deployed outdoors [74, 79–81].

In a recent study in the lower Jordan Valley, Attractive Sugar Baits (ASB) laced with the mosquito biocontrol aerobic bacterium *Bacillus sphaericus* were used to suppress *An. sergentii* populations [82]. The suppressive effect was achieved by adults contaminating larval breeding sites with *B. sphaericus*, resulting in larval suppression rather than a direct effect on the lifespan of adults [82]. This, and other compounds that target blood feeding insects specifically, will be key to the acceptance of ATSB as a broadly applicable novel intervention tool. Indeed, modelling studies have demonstrated the potential power of deploying ATSBs, particularly in combination with existing interventions for control of malaria vectors in SSA regions hyperendemic for malaria [77, 83].

## Larvicides

Targeting the immature stages of malaria vectors in their outdoor aquatic habitats is increasingly being considered as an arm required for achieving malaria elimination in sub-Saharan Africa. This method of control was the cornerstone of several malaria control programmes and was used with greatest success in the eradication of invasive populations of *An. gambiae* and *An. arabiensis*, in Egypt and Brazil respectively [84, 85]. Due to widespread resistance to some chemical compounds and their toxicity within the environment, biolarvicides are the preferred choice, because they make use of toxic proteins produced naturally in some soil bacteria. Large-scale application of the biolarvicide *Bacillus thuringiensis* var. *israelensis* (Bti) in Burkina Faso over three years resulted in a dramatic decrease in exposure to bites [86]. Across SSA ecosystems, larval control using Bti and *Bacillus sphaericus* (Bs) combined with ITNs resulted in significant decreases in malaria vector densities which translated in a decrease in malaria transmission in some but not all areas [87–89]. These mitigated results illustrate the difficulties inherent in identifying and treating numerous ephemeral *Anopheles* vector breeding sites with larvicides that have a short duration of activity [90]. The limitations can make larval control laborious and costly. Long-lasting microbial larvicides FourStar briquets (Central Life Sciences, Sag Harbor, NY, USA) and LL3 (University of California, Irvine, CA, USA) were developed to surmount low residual activity. The use of FourStar in Kenya significantly reduced indoor and outdoor biting by malaria vectors [91]. In the same country, combined FourStar and LL3 applications significantly reduced all stages of *An. gambiae* and *An. funestus* larvae densities for up to 20 weeks compared to a non-intervention area [92] with no significant impact on non-target organisms [93]. To avoid resistance to these biolarvicides other biological control interventions have been proposed. For example, laboratory and field tests conducted in Benin showed that treatment of larval breeding sites with eggs of the nematode *Romanomermis iyengari* significantly reduced *An. gambiae* larvae density [94]. Early larval stages are more susceptible to infection, hence nematode control should be applied shortly after rainfall and relies on extensive surveying of breeding sites. Interestingly, nematode applications targeting the South American malaria vector *An. albimanus* in Colombia, resulted in decreased larval densities and malaria prevalence in children [95].

Importantly, biolarvicides can impact vector populations irrespective of their level of resistance to pesticides and degree of endophily. They can specifically target anophelines, resulting in fewer effects on non-target organisms than with chemical larvicides. Therefore, and provided that the frequency of their application in different ecological settings can be effectively managed, larval biocontrol offers much promise for integrated vector control programmes in SSA.

## Genetic vector control approaches

Mosquito release programmes that rely on the release of sterile male, genetically-modified mosquitoes, or mosquitoes carrying a genetically-modified symbiont offer a completely different approach to control anopheline vector populations, which importantly, is independent of their degree of endophagy, endophily, timing of biting and anthropophily.

### Sterile mosquito releases

The oldest of the so-called genetic vector control approaches is the sterile insect technique (SIT), which has been used since the 1950s as a species-specific and environmentally-friendly method of controlling insect populations [96]. It relies on mass-rearing of males that are sterilised by irradiation or chemicals and released in large numbers into the mapped-out area [97]. Wild females that mate with sterile males do not produce viable offspring. SITs have been successfully deployed against a variety of insect pests but have so far had limited success against mosquito vector control [98–108]. For the control of large and complex African malaria vector populations, SIT is usually not considered a realistic strategy due to the large scale of releases required [109]. For this reason, ongoing programmes targeting African malaria vectors focus only on small and/or ecologically-isolated populations such as *An. arabiensis* in Northern Sudan or KwaZulu, Natal in South Africa [110–113]. These projects are in their developmental stages and have generated the first data on the survival, dispersal and mating competitiveness sterile males, all crucial components for determining adequate male release ratios. A drawback of classic SIT is that radio-sterilization negatively affects male mating competitiveness, and this has been confirmed in *An. coluzzii* [114] and *An. arabiensis* [110]. Despite this, small-scale releases in Sudan, showed that irradiated *An. arabiensis* males participated in natural swarms, suggesting that inundative releases could be effective for local control strategies. Currently, the actual mating success of males remains to be determined [112]. The paucity of these examples highlights the urgent need for research focusing on the ecology of malaria vectors mosquito releases.

SIT programmes require extensive infrastructure and typically need to be sustained for long periods of time to negate the effects of re-invasion by migrant mosquitoes, and this constrains their cost effectiveness. However, the current context of decreasing vector densities observed in parts of Africa may increase the scope for controlling residual malaria vector populations with self-limiting SIT-like interventions.

### Genetically modified mosquitoes

Nowadays, sterile males can be created by molecular engineering, removing the need for radio- or chemical sterilisation. GM and SIT sterile male releases have the same reliance on mass production and inundative releases and are thus not considered a scalable strategy for the control of large complex SSA anopheline populations. However, other genetic-modification approaches exploit the principle of genetic inheritance to introduce and spread epidemiologically relevant effector genes into mosquito populations. In population replacement strategies, the introduced gene may, for example, interfere with a vector’s capacity to support development and transmission of pathogens resulting in a refractory population. In population suppression approaches, the genetic modification is designed to decrease the fertility of female mosquitoes or the sex-ratio of their progeny resulting in population crash [115, 116].

Genetically modified mosquitoes are an increasingly promising prospective tool for integrated vector management. Over the past decade, genetic approaches have benefited from major innovations in genetic engineering, but their future deployment is contingent on broad public and regulatory acceptance is therefore currently much more complex compared to SIT interventions. At the technical level, the biggest challenge initially faced by GMM vector control approaches stemmed from the fact that the spread of effector genes through wild populations was constrained by Mendelian inheritance and fitness costs associated with genetic modifications [117, 118]. The recent development of gene drives that bypass Mendelian inheritance has resolved these issues [119]. A number of recent laboratory studies have confirmed that genes conferring refractoriness to pathogens or genes suppressing mosquito populations by affecting female fertility or creating sex-ratio distortion can effectively spread through anopheline populations [120–124]. Modelling studies have also shown the strong potential impact of such intervention on vector dynamics under a wide array of environmental conditions [125].

Gene drives take advantage of selfish genetic elements such as homing endonuclease genes (HEGs) that can recognize and cleave a specific DNA target site of 20–30 bp [126]. The cell’s DNA repair machinery allows the HEG being copied on the homologous chromosome, *via* homology directed repair (HDR), to be spread in super-Mendelian fashion over subsequent meiotic events and generations. In *An. gambiae* (*s.s.*), Windbichler et al. [127] showed that a HEG inserted in an autosomal locus could spread and knockout a synthetic gene expressing a fluorescent marker through mosquito caged populations. In 2016, CRISPR-Cas9 was used to knockout genes responsible for *An. gambiae* female fertility showing a capacity to spread over consecutive generations. However, genetic resistance impeded the complete suppression of the caged populations [122, 128]. In *An. stephensi*, an autosomal drive based on CRISPR-Cas9 and HDR mechanism was developed to spread anti-*Plasmodium falciparum* molecules [124]. Another strategy uses endonuclease genes to cleave X-linked rDNA sequences during spermatogenesis resulting in male-biased sex-distortion and population suppression when released at high rates in a caged population [121, 122]. The insertion of the “X-shredding” construct on the Y chromosome using CRISPR-Cas9 resulted in stronger male bias and drive [121]. Similarly, chemical vector control approaches, genetic modifications aiming to achieve population suppression or replacement, are vulnerable to possible evolution of resistance mechanisms. This is now taken into consideration at the genetic engineering level and is also being investigated through simulation models [129, 130]. Recently, CRISPR-Cas9 was used to target a highly conserved and functionally constrained DNA sequence within the *double sex* gene, responsible for *An. gambiae* sex differentiation, resulting in the rapid spread of the genetic knockout and population crash without selection of genetic resistance in the laboratory [123]. As is the case for chemical control, another possible solution to manage the emergence of resistance would be the deployment of several variants of gene-drive strains. Therefore, the ability to produce strains with multiple effector mechanisms or multiple strains with contrasted molecular effector processes may be key and requires consideration early on. Another limitation stems from the fact that this approach currently requires genetic introgression of the driving constructs into locally-colonised wild-type genetic backgrounds which is not always feasible. Finally, genetic approaches face considerable challenges in terms of public perception and regulatory requirements [131, 132].

### Paratransgenic approaches

Instead of relying on engineered mosquitoes, other population replacement approaches focus on modifying symbionts within mosquitoes. One such approach consists in colonizing mosquitoes with genetically modified symbiotic organisms such as bacteria, viruses and fungi, able to express effector molecules in order to achieve an antibiosis relationship towards the pathogens they transmit [133–135]. Another strategy aims to modify symbionts resulting in imbalance in mosquito microbiome, which, in turn, results in reduced lifespan, hence vectorial capacity [136, 137]. With that in mind, detailed studies have described mosquito bacterial communities and bacteria displaying important roles in mosquito biology, including mosquito-pathogen interactions [136, 138–143]. Symbiotic bacteria species of the genera *Asaia*, *Serratia* and *Panthoea* produced promising outcomes by significantly decreasing *Plasmodium* prevalence in anophelines [137, 144–147]. The absence of fitness costs in terms of mosquito longevity and fecundity [137, 145, 147] is paramount for the transmission of genetically modified (GM) bacteria in subsequent generations. Successful vertical and horizontal transmission experiments of GM *Asaia* in semi-field experiments demonstrated ability to spread engineered symbionts in mosquito populations making paratransgenesis a promising new tool for controlling vector-borne diseases. In parallel to those efforts, Cirimotich et al. [148] have isolated natural bacteria species in wild populations of *An. arabiensis* that inhibited the development of *P. falciparum*. However, the potential of this approach for vector control needs to be further explored.

## *Wolbachia* releases

The endosymbiotic bacteria, *Wolbachia* colonises the gonads of many insect species and can promote its spread through its host populations *via* cytoplasmic incompatibility [149]. In mosquitoes, *Wolbachia* can also negatively affect the development of viruses and pathogens [150]. These characteristics have led to the development and implementation of strategies in which cytoplasmic incompatible *Wolbachia*-carrying strains are mass-reared and released for the control of arbovirus transmission [149, 150]. The potential use of *Wolbachia* infection for preventing *Plasmodium* transmission in anopheline populations is a particularly exciting perspective [151]. Experimental studies in *An. gambiae* have reported that *Wolbachia* infection can induce an upregulation of immune genes that can inhibit *Plasmodium* development [152, 153]. However, in contrast to what is observed in *Aedes aegypti*, the prevalence and transmission of *Wolbachia* in natural populations of the malaria mosquito *An. gambiae* are much lower, which currently hinders the development of such strategy for malaria control [152, 153]. Further research is therefore urgently needed to boost the prospects of *Wolbachia*-infected anophelines release towards malaria vector control.

## Endectocides

The recent discovery that ivermectin antifilarial drug treatments were also active against ectoparasitic infestations such as lice and scabies [154, 155] opened up another novel strategy for the control of anopheline vectors. Treating human hosts or their domestic animals with molecules that can reduce the density of the insects that feed on them is an approach that would be equally effective against indoor and outdoor mosquito populations [156]. In Burkina Faso, Pooda et al. [157] reported an increase in mortality and decrease in fertility of *An. coluzzii* feeding on cattle treated with ivermectin. Interestingly, in Senegal, mass ivermectin treatment of the human population in three villages, negatively affected the longevity of blood-fed *An. gambiae* females [158]. In a larger study focusing on mass drug administration of ivermectin in three countries of West Africa, a significant decrease in longevity was recorded which translated in lower sporozoite rates in both indoor resting and outdoor host-seeking *An. gambiae* (*s.l*.) populations [159]. Ongoing trials focus on balancing the need for high doses of ivermectin required to maintain adequate mosquitocidal activity with possible side effects [160]. Other research efforts are seeking to find alternative and longer lasting compounds that could be used towards mass-drug administration strategies [161].

## Conclusions

There are a large number of tools available for malaria vector control, some proven and tested, some being refined and others in development stages. So far, the few affordable and scalable tools endorsed by WHO and deployed by RBM have targeted indoor biting vector populations. These interventions are losing effectiveness by the day and are no longer adequate in many settings where malaria transmission is now significantly sustained by outdoor biting vector populations. The spread of insecticide resistance in malaria vectors and the shift in vector composition and feeding pattern resulting from sustained selection pressure on endophilic mosquitoes calls for additional control tools dealing specifically with such increasingly common phenotypes. In this review, we highlight some of the existing or emerging tools which may be particularly effective for surveillance and control of outdoor biting malaria vectors. Whilst this list might seem long, there are truly few approaches that combine cost effectiveness, scalability and sustainability. The recent development of synthetic attractants for counter-flow traps have shown encouraging results for ongoing-malaria surveillance and monitoring but their cost is an obstacle to scalability in rural settings. The use of larvicides, perhaps combined with novel models of deployment *via* communities and/or technologies, may be feasible in urban and semi-urbanised settings. Amongst the truly novel tools, sugar baits and endectocides could provide cost effective and scalable angles of attack for the control of outdoor-biting malaria vectors and offer versatility in the way that they can be dispensed in various settings. Finally, advances in genetic engineering and modelling of gene-drives for vector population suppression or replacement offers new ways of targeting malaria vectors with fast changing biting behaviour. It is hoped that a more diverse toolbox will facilitate increased versatility and integration of vector control management, as well as adopting more responsible and sustainable use of classic chemical control tools.

## Data Availability

The datasets generated and/or analysed during the present study are available from the corresponding author on reasonable request.

## Abbreviations

ITNs: Insecticide-treated nets
IRS: Indoor residual spraying
WHO: World Health Organization
RBM: Roll Back Malaria
SSA: Sub-Saharan Africa
PSC: Pyrethroid spray catch
CDC-LT: Centre for Disease Control light traps
HLC: Human landing catches
CFG: Counter flow geometry
BGS: Biogent Sentinel
BGM: Biogent malaria
MM: Mosquito magnet
MET: Mosquito electrocuting trap
RB: Resting boxes
SRB: Sticky resting boxes
ATSB: Attractive toxic sugar baits
SIT: Sterile insect technique
GMM: Genetically modified mosquitoes
HEG: Homing endonuclease genes
HDR: Homology directed repair
